# Case report: Three-dimensional printing as an educational tool for optimal lead positioning to left bundle branch pacing

**DOI:** 10.3389/fcvm.2022.973480

**Published:** 2022-09-15

**Authors:** Hui-Qiang Wei, Yumei Xue, Shulin Wu, Xianhong Fang

**Affiliations:** Department of Cardiology, Guangdong Cardiovascular Institute, Guangdong Provincial People's Hospital, Guangdong Academy of Medical Sciences, Guangzhou, China

**Keywords:** left bundle branch area, physiological pacing, 3-dimensional printing, interventricular septum, pacing lead

## Abstract

Left bundle branch pacing (LBBP) has been widely adopted as a physiological pacing approach. However, LBBP fails to achieve in some cases because it is difficult to maintain the orientation of the lead tip perpendicular to the interventricular septum (IVS). Three-dimensional (3D) printing technology has emerged as a promising tool for modeling and teaching cardiovascular interventions. Seeking confirmation of optimal lead placement relative to the IVS, we used 3D printing technology to generate a 3D printed heart from a selected patient with successful and proven LBBP. Our model successfully illustrated that the lead tip was perpendicular to the IVS. Application of the 3D technology has potential to help the early-operator understand the optimal lead placement relative to IVS and diminish the learning-curve.

## Introduction

Medical 3-dimensional (3D) printing has been applied to cardiovascular diseases in recent years (1). 3D printing has become a promising tool for modeling and teaching cardiovascular interventions. Left bundle branch pacing (LBBP), a new physiological pacing strategy, is considered as a feasible and safe approach characterized by a narrow QRS duration and low and stable capture threshold (2). Ideally, the pacing lead is screwed-in perpendicular to the interventricular septum (IVS) during the implantation procedure (3). However, LBBP cannot be achieved in some cases because it is difficult to maintain the orientation of the lead tip perpendicular to the IVS. To better examine the position of the lead relative to the IVS, we reviewed clinically indicated cardiac computed tomography (CT) scan of a patient with LBBP lead and generated 3D-printed model.

## Case report

**Figure 1:**
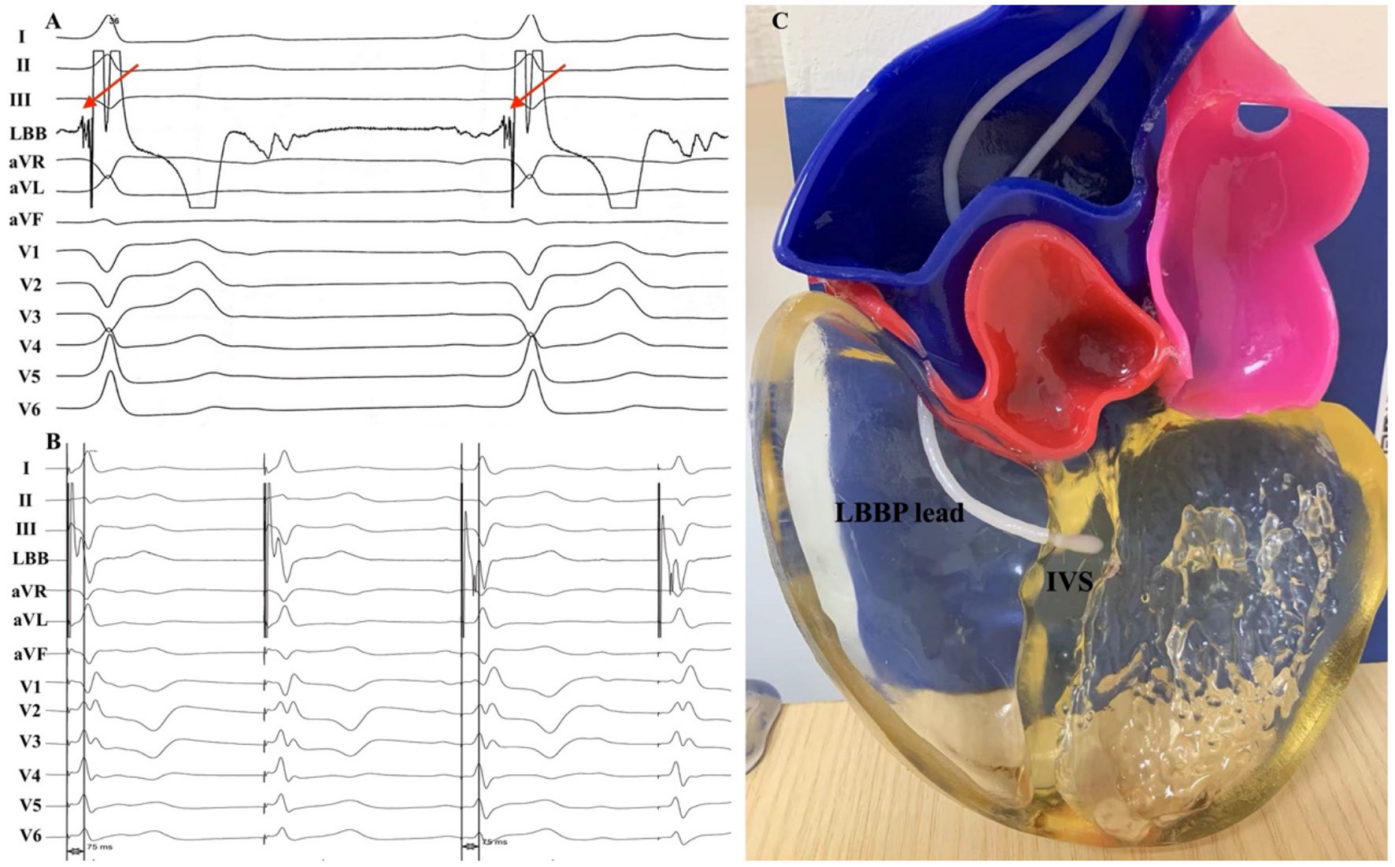
**(A)** 12-lead ECG and unipolar tip electrode electrogram obtained during sinus rhythm and **(B)** LBBP. **(C)** Three-dimensional printed model. **(A)** A LBB potential (red arrow) was recorded during the implant procedure. **(B)** During the threshold test, a transition from non-selective to selective LBBP was observed with a stable peak LV activation time of 75 ms. LBB, left bundle branch; LBBP, left bundle branch pacing; LV, left ventricular; IVS, interventricular septum.

A 61-year-old male presented with recurrent syncope and was found to have high-degree atrioventricular block. He was admitted to our institution for pacemaker implantation. We intended to place the ventricular lead in the left bundle branch (LBB) area to deliver physiological pacing. The 3830 pacing lead (SelectSecure, Medtronic, Minneapolis, MN, USA) was delivered through a fixed-curve sheath (C315 His, Medtronic, Minneapolis, MN, USA) inserted *via* the left axillary vein. Then the sheath and the lead were advanced to the ventricular side inferior to the septal leaflet of tricuspid valves and rotated in a counterclockwise fashion to place the lead tip in a perpendicular orientation toward the IVS. The pacing lead was successfully placed in the LBB area and LBB potential was recorded with a low capture threshold of 0.75V/0.5ms (Figure 1A). The transition from non-selective LBBP to selective LBBP was recorded with the same peak LV activation time of 75 ms (Figure 1B). No complications occurred during the procedure.

Cardiac CT was performed in this patient due to the chest pain evaluation. CT scans were obtained with a 64-slice spiral CT system (GE Healthcare, Milwaukee, WI, USA), with retrospective gating and a slice thickness of 0.75 mm. Then 3D reconstruction of the heart was performed using the Mimics software (Materialize NV, Leuven, Belgium). Model was printed using the J501Pro printer (Zhuhai Seine Technology Co., Ltd., Zhuhai, China) with photosensitive resin. The printing procedure took ~5 h to complete. The result of the model is shown in Figure 1C.

## Discussion

LBBP has been widely used in clinical practice since it was initially described by Huang in 2017 (4). Several methods for guiding LBBP lead implantation have been reported (5, 6). However, the failure rate of LBBP was 10–20% (7). One of possible explanations may be related to the oblique lead fixation. 3D printing technology is a promising tool for modeling and teaching cardiovascular interventions. Patient-specific 3D models may offer useful anatomic information and guide the operators how to rotate the sheath. The example of our 3D printing model as an educational tool for optimal lead positioning to LBBP showed that the lead tip was perpendicular to the IVS. The LBB potential was recorded during the procedure and selective LBBP was successfully achieved in this patient. Application of the 3D technology has potential to help the early-operator understand the optimal lead placement relative to IVS and diminish the learning-curve.

## Conclusion

The 3D printed model may help to better understand the relationship between the lead and IVS. Future applications of 3D printing might include facilitating research focusing on optimal lead positioning, understand complex anatomy and plan complex implantation procedure.

## Data availability statement

The raw data supporting the conclusions of this article will be made available by the authors, without undue reservation.

## Ethics statement

The studies involving human participants were reviewed and approved by Guangdong Provincial People's Hospital. The patients/participants provided their written informed consent to participate in this case study. Written informed consent was obtained from the individual(s) for the publication of any potentially identifiable images or data included in this article.

## Author contributions

All authors listed have made a substantial, direct, and intellectual contribution to the work and approved it for publication.

## Funding

This work was supported by the Science and Technology Planning Program of Guangdong Province (grant number 2019B020230004).

## Conflict of interest

The authors declare that the research was conducted in the absence of any commercial or financial relationships that could be construed as a potential conflict of interest.

## Publisher's note

All claims expressed in this article are solely those of the authors and do not necessarily represent those of their affiliated organizations, or those of the publisher, the editors and the reviewers. Any product that may be evaluated in this article, or claim that may be made by its manufacturer, is not guaranteed or endorsed by the publisher.

## References

[1] Wang DD Qian Z Vukicevic M Engelhardt S Kheradvar A Zhang C. 3d printing, computational modeling, and artificial intelligence for structural heart disease. JACC Cardiovasc Imaging. (2021) 14:41–60. 10.1016/j.jcmg.2019.12.022 32861647

[2] Vijayaraman P Subzposh FA Naperkowski A Panikkath R John K Mascarenhas V. Prospective evaluation of feasibility and electrophysiologic and echocardiographic characteristics of left bundle branch area pacing. Heart Rhythm. (2019) 16:1774–82. 10.1016/j.hrthm.2019.05.011 31136869

[3] Huang W Chen X Su L Wu S Xia X Vijayaraman P. A beginner's guide to permanent left bundle branch pacing. Heart Rhythm. (2019) 16:1791–6. 10.1016/j.hrthm.2019.06.016 31233818

[4] Huang W Su L Wu S Xu L Xiao F Zhou X. A novel pacing strategy with low and stable output: pacing the left bundle branch immediately beyond the conduction block. Can J Cardiol. (2017) 33:1736 e1–3. 10.1016/j.cjca.2017.09.013 29173611

[5] Jiang H Hou X Qian Z Wang Y Tang L Qiu Y. A novel 9-partition method using fluoroscopic images for guiding left bundle branch pacing. Heart Rhythm. (2020) 17:1759–67. 10.1016/j.hrthm.2020.05.018 32417259

[6] Liu X Niu HX Gu M Chen X Hu Y Cai M. Contrast-enhanced image-guided lead deployment for left bundle branch pacing. Heart Rhythm. (2021) 18:1318–25. 10.1016/j.hrthm.2021.04.015 33887449

[7] Li Y Chen K Dai Y Li C Sun Q Chen R. Left bundle branch pacing for symptomatic bradycardia: implant success rate, safety, and pacing characteristics. Heart Rhythm. (2019) 16:1758–65. 10.1016/j.hrthm.2019.05.014 31125667

